# Safety and Academic Outcomes of College Campus-Based Advocacy Services

**DOI:** 10.1177/08862605231198487

**Published:** 2023-10-21

**Authors:** Rachel J. Voth Schrag, Elizabeth Baumler, Dixie Hairston, Cynthia Jones, Leila Wood

**Affiliations:** 1 The University of Texas Arlington, USA; 2 University of Texas Medical Branch, Galveston, TX, USA; 3 The University of Texas Rio Grande Valley, Edinburg, USA

**Keywords:** dating violence, domestic violence, sexual assault, stalking, intervention, intervention/treatment

## Abstract

Intimate partner violence (IPV), sexual assault, and stalking are consequential public health and safety issues with wide reaching impacts on emerging adults, including those on college campuses in the United States. In response to high rates of violence among college student populations, universities are developing campus-based advocacy (CBA) programs, which aim to support survivors of interpersonal violence through supportive connections, resource acquisition, and safety planning. However, little data exists related to their impact on key student-survivor outcomes. Thus, this study aims to understand (a) the approach CBA programs use to address safety and academic concerns of student-survivors, and (b) the initial outcomes of CBA programs on safety and academics among students engaged in CBA services at five universities in one Southwestern state. The project used a longitudinal mixed-methods approach, with data collection activities including qualitative interviews with student survivors (*n* = 29) and a longitudinal, web-based, quantitative survey with matched analyses of safety and academic outcome measures from 115 student survivors who participated in an initial survey and follow-up survey after 6 months. Findings demonstrate key pathways through which CBA programs support survivors and facilitate positive safety and academic outcomes. These pathways include education, supportive connection, and resource access. Analysis of longitudinal survivor data demonstrate substantial reductions in sexual violence, IPV, stalking, and school sabotage at 6-month follow-up compared to initial survey, as well as significant reductions in academic disengagement for student survivors. The findings of the study powerfully demonstrate the positive impact of CBA programs on survivor and campus outcomes. Furthermore, programs not only enhance individual survivor safety and academic outcomes but also support the overall climate and safety of hosting universities.

## Introduction

Intimate partner violence (IPV), sexual assault, and stalking are consequential public health and safety issues with wide reaching impacts on emerging adults (ages 18–25), including those on college campuses in the United States. Multi-institution studies conducted over the last decade have illustrated the high prevalence of these forms of violence with IPV rates ranging from 7% to 25% (Busch-Armendariz et al., 2017; Cantor et al., 2020; Krebs et al., 2016; Munro-Kramer et al., 2021) stalking rates of 5.8% to 13% (Busch-Armendariz et al., 2017; Cantor et al., 2020) and sexual assault rates of 13% to 25% (Cantor et al., 2020; Krebs et al., 2016; Mellins et al., 2017). Tactics of school sabotage, a form of IPV impacting academics, were found in 20% of the most recent relationships in a sample of community college students in the United States (Voth Schrag et al, 2020). Interpersonal violence has long-lasting impacts in the lives of college students, especially related to risks for revictimization (Wood et al., 2021; Mellins et al., 2017; Walsh et al., 2020) and academic engagement (Wood et al., 2018; Potter et al., 2018). College campuses have responded to the express needs of survivors in part through the development and implementation of campus-based advocacy (CBA) programs to provide supportive services to survivors, with a specific focus on IPV, sexual assault, and stalking (Wood et al., 2021; Javorka & Campbell, 2019; Klein et al., 2016). CBA services are adapted from community-based service models and focus on safety, resources, and community supports for survivors (Wood et al., 2021; Klein et al., 2023). Community-based advocacy services, such as those in IPV and sexual assault focused non-profits, have been demonstrated repeatedly to increase survivor safety, reduce repeated victimization, increase economic stability, and improve health (Rivas et al., 2015; Sullivan, 2021). While advocacy programs are becoming increasingly prevalent on college campuses, very little data exists that demonstrates their impact on student-survivor outcomes. Thus, the current study employed a longitudinal mixed-methods approach to understand the safety and academic outcomes of students using CBA services at five universities in a Southwestern state. The study sought to understand (a) the approach CBA programs use to address safety and academic concerns and (b) changes in safety and academics experienced by CBA participants over 6 months.

## Background

Emerging adulthood is a time of increased risk for interpersonal violence victimization (Wood et al., 2021; Coker et al., 2016), meriting a focus on students in collegiate settings. Driven by student activism, increased research, and changing policy landscapes, the last decade has seen an increase in assessments of campus climate and prevalence of violence, research on college violence prevention programs, and attention to the help-seeking needs of student survivors (McMahon, 2018; Moylan et al., 2022; Swartout et al., 2018). Student-survivors of IPV, sexual assault, and stalking on college campuses face a range of negative consequences, including disruptions to housing and economic stability, trauma symptoms, increased substance misuse, peer and social support disruptions, increased isolation, and physical health impacts (Voth Schrag et al., 2020; Wood et al., 2018; Waterman et al., 2019). While IPV, sexual assault, and stalking occurs in all student groups, women, undergraduates, BIPOC students, international, and LGBTQ students may have heightened risks (Busch-Armendariz et al., 2017; Krebs et al., 2016).

Among the most insidious impacts of violence is academic disengagement, including behaviors that lead to withdrawal from academic pursuits, such as dropping out, not attending class or completing course work, as well as behaviors that disrupt academic attainment, such as coming to class under the influence of substances (Kaukinen, 2014). Previous studies have shown that experiencing interpersonal violence in college can lead to disruption in education trajectories including decreased grades, transferring, changing majors, or leaving higher education all together (Voth Schrag et al., 2020; Potter et al., 2018). Experiences of interpersonal violence are known to increase survivors’ risk for future violence victimization, perpetrated by the same or a different individual, and across forms of victimization (Wood et al., 2021; Walsh et al., 2020). Substance use and negative mental health symptoms are all contributors to the likelihood of revictimization (Mellins et al., 2017; Walsh et al., 2020). Services for survivors that focus on increasing immediate safety, harm reduction behaviors, and increasing empowerment may decrease the risk of revictimization (Cusak et al., 2019; Walsh et al., 2020). Despite this increased attention to interpersonal violence in higher education, little research has been conducted on the impact of services provided to survivors on college campuses.

### CBA Services

CBA services provide supports to survivors of IPV, sexual assault, stalking, and other types of harm or violence. These services, like community-based advocacy for interpersonal violence survivors, are typically based in tenants of trauma-informed care, social justice, and empowerment theories (Wood et al., 2020, 2022) and use a student-survivor centered model (Voth Schrag et al., 2020; Klein et al., 2023). Advocacy typically uses survivor-centered, trauma-informed, low-barrier, and voluntary approaches to address safety, health, and economic needs (Wood et al., 2020; Nnawulezi et al., 2018). Advocates aim to enhance both the internal capacity for survivors to address and preempt experiences and impacts of abuse, as well as help to build communities of support and safety around survivors (Goodman et al., 2017). Adapted for campuses, CBA models seek to address expressed needs in a confidential, developmentally appropriate, and culturally relevant way (Wood et al., 2021; Javorka & Campbell, 2021). Advocacy in the campus setting may deviate from community-based programs by focusing on academic needs, the developmental tasks of emerging adulthood, and consideration of reporting implications of policies such as Title IX (Wood et al., 2021; Brubaker & Keegan, 2019; Klein et al., 2023). Programs are offered in a range of setting, including as a standalone unit, embedded in a community program, or in partnership with other campuses services (Klein et al., 2023) such as law enforcement, counseling, student health, or Title IX (Javorka & Campbell, 2021).

CBA programs typically address safety and academic needs, two top concerns of survivors in college settings (Wood et al., 2021). Much like community-based counterparts, CBA staff focus on individualized safety planning with students, identifying and addressing immediate specific risks faced by students in order to reduce the extent and impact of violence (Davies & Lyon, 2014; Messing et al., 2015). CBA staff engage with students in the process of *academic safety planning*, developing a personalized practical plan to address physically and emotionally dangerous situations that might impact a student’s academic achievement or engagement, as well as developing a plan for obtaining accommodations and addressing choices around institutional processes in a student-centered manner (Voth Schrag et al., 2020). Advocacy on campus also addresses a broad range of safety needs via resource provision and information. Programs address immediate physical safety needs through helping students identify and move into safe living situations, developing plans for safe navigation around the campus context, and identifying strategies for safety in online environments (Wood et al., 2021; Javorka & Campbell, 2021). CBA programs address emotional safety via supportive listening, identification and support for coping strategies, and provision or referral to mental health support (Wood et al., 2021). CBA programs support both academic needs and safety impacts of violence through assisting with accommodations, changing schedules or class/lab times, and liaising with faculty and other institutional actors to support students choices and safe engagement in school (Wood et al., 2021).

### The Current Study

CBA programs are expanding in universities across the United States in order to address the unique needs of students (Klein et al., 2023). However, little is known about how these programs impact survivor safety and academic experiences, although these are critical outcomes for collegiate survivors. To assess the routes through which the CBA service model may impact safety and academics, this study employed a longitudinal mixed-methods approach to capture the experiences of student-survivors who have used CBA services in programs at five universities in a diverse Southwest state. Study research questions include (a). How do campus advocacy programs address survivor safety and academic concerns? And (b). What changes are observed in safety and academics for student-service users over 6 months?

## Methods

Data for this study come from an evaluation conducted across CBA programs at five public universities in one southwestern state in the United States. The larger project aimed to define and evaluate the CBA service model, understand service user experiences, and identify evaluation strategies that maybe used to support program growth. More information on the larger study can be found in the ***Campus Based Advocacy ToolKit*** (Wood et al., 2021). For the current study assessing program outcomes, longitudinal repeated surveys (*n* = 115) and follow-up interviews (*n* = 29) were analyzed. Data were collected for the current study from August 2019 to August 2021. All study activities were approved by the institutional review board of the sponsoring university prior to the beginning of data collection. The participating programs all offered advocacy services separate from university Title IX services. All the campuses were part of one university system, with student populations ranging from 23,000 to 50,000 students.

### Procedures

CBA service users from all five campuses were recruited with the assistance of partner programs. Programs distributed information about the survey to students who had utilized advocacy service in the previous 6 months via promotional e-mail or secure message with a survey link, project information, and contact information for the study team. Potential participants were eligible for the study if they had participated in advocacy, or support services, related to experiences of sexual assault, sexual harassment, stalking, or dating violence in the past 6 months and were 18 years old or older at the time of data collection. A total of 134 students from the five participating programs initially enrolled in the longitudinal evaluation completed the initial survey and gave consent to the study team to be recontacted for follow-up data collection, and 115 were retained at 6-month follow-up and included in matched (baseline-follow-up) analyses, representing a 6-month retention rate of 80.6%. The study team communicated with participants via their preferred, safe forms of communication (text, phone, or email). Participants were contacted for follow-up survey assessments at 3 and 6 months past their initial impact survey. Survey assessments included standardized measures of program experiences and associated outcomes developed from an earlier process evaluation (Wood et al., 2021). Data from both the initial survey and 6-month follow-up are presented in this study. Participants received a $10 gift card for the initial impact survey and $15 for each follow-up survey as a thank you.

### Measures

#### Safety-Related Empowerment

To capture the extent of participants empowerment related to safety, the study team adapted two subscales from the Measure of Victim Empowerment Related to Safety (MOVERS), from Goodman et al. (2015). Adaptations were designed to address the campus context. To assess participants’ sense of their ability to navigate their own safety decisions, five items were adapted from the internal tools subscale (e.g., *I know what the next steps are in my path towards staying safe at school*). The internal reliability for the adapted scale in this sample, measured by alpha (Cronbach), is.87. To capture the extent to which participants expect their campus community to be able to provide useful support as they address experiences of interpersonal violence, the study team adapted four items from the expectations of support subscale (e.g., *Campus programs and services provide support I need to keep safe).* Alpha for the adapted expectations of support scale in this sample is 0.84. All MOVERS items are measured from Not at all true (1) to Very True (4).

#### Sexual Assault

Extent of sexual assault victimization was assessed using 4 items adapted from the Sexual Experiences Survey (SES; Koss et al., 2007). Response options indicated the frequency of experiences, from none (1) to 3+ times (4). The included items were: (a) someone fondled, kissed, or rubbed up against the private areas of my body (lips, breast/chest, crotch, or butt) or removed some of my clothes without my consent (but did not attempt sexual penetration); (b) someone had oral sex with me or made me have oral sex with them without my consent; (c) someone put their penis, fingers, or other objects into my vagina without my consent, and (d) someone put their penis, fingers, or other objects into my butt without my consent. Participants were considered to have experienced sexual violence if they endorsed at least one item. The SES is an established scale with strong reliability, and an established alpha of .80.

#### Stalking

Extent of stalking victimization was assessed using seven items adapted for the study from the National Intimate Partner and Sexual Violence Survey (NISVS) stalking questioner (Smith, 2018), which captures physical and digital stalking behaviors. Response options indicated the frequency of specific experiences, ranging from none (1) to more than eight times (5). Participants were considered to have experienced stalking if they endorsed experiencing two or more behaviors. These items have an established Cronbach’s alpha of .73.

#### Intimate Partner Violence

Experiences of IPV were assessed using six items adapted from the Partner Victimization Scale (Hamby, 2013). Response options included the frequency of specific experiences during the time period in question ranging from never (1) to many times (6). Participants were considered to have experienced IPV if they endorsed experiencing at least one behavior. Cronbach’s alpha in this sample was .82, indicating strong internal reliability.

#### School Sabotage

Experiences of school sabotage were assessed using eight items adapted for the study from the School Sabotage Scale (Voth et al., 2020). Response options included the frequency of specific experiences such as physical violence at school, disrupting homework, or sabotaging financial aid, during the time period in question, ranging from never (1) to many (6) times. Cronbach’s alpha in this sample was .87, indicating strong internal reliability.

#### Academic Disengagement

The impact of violence on engagement with school was assessed using an academic disengagement measure consisting of 11 items adapted from (Hanisch & Hulin, 1990; Ramos, 2000), querying the frequency of disengagement behaviors due to violence from 1 (never) to 5 (always). Participants were asked how frequently they experienced academic outcomes due to violence or abuse from enrollment to initial survey or from initial survey to follow-up. Example items include *Missed class because of abuse, violence, or harassment experiences, turned in poor schoolwork, dropped a class, thought about quitting school.* Cronbach’s alpha in this sample was .87.

#### Grade Point Average

Two additional items queried student grade point average (GPA). Students were asked to self-report their current GPA (A = 1, B = 2, etc.). Students were also asked if this GPA is higher, lower, or about the same as the previous semester.

### Qualitative Interviews

Participants enrolled in the longitudinal study were invited for a follow-up interview, with an aim to collect data from students across partner campuses and with different levels of violence experience at follow-up. A total of 29 semi-structured interviews were conducted via Zoom, phone, or in person with student service users. At the end of each impact survey, participants were asked if the research team could reach out to them about further research. Participants who consented to being contacted again were sent an email requesting a follow-up interview. All interviews were voluntary, confidential, and conducted by study team members with experience conducting qualitative interviews with survivors of violence. Interviews lasted 45 to 60 min on average and were audio recorded and professionally transcribed verbatim for analysis. Interview participants were provided a $20 gift card for their participation. The interview protocol included questions such as “What happened at your first experience with [program]?” “What is the most important service you received from [program]” “How did [program] impact your academic experience?” and “How did [program] impact your safety needs?” The full interview guide, which was amended for these interviews, can be found in [open access resource, blinded].

### Participants

One-hundred fifteen service users who participated in the initial impact survey and were retained at follow-up are included in this analysis (see Table 1). At the initial survey, half of participants were juniors and seniors (50.3%), with others identifying as freshman, sophomores, and graduate students. Most were living off campus in their own housing, with approximately 20% living in campus owned housing either on or off campus. Most identified as female (*n* = 107), and the sample came from diverse racial/ethnic backgrounds including Hispanic/Latine, white, Asian, and Black/African American. The majority of participants identified their sexual orientation as heterosexual/straight, with others identifying as bisexual, or another sexual orientation.

**Table 1.** Participant Demographics.

Longitudinal Survey Participants (*n* = 115)		Interview Participants (*n* = 29)	
	%(*n*)		%(*n*)
Race/ethnicity		Race/ethnicity	
Hispanic/Latine	30.6% (39)	Hispanic/Latine	52.7% (15)
White	32.3% (40)	White	27.6% (8)
Black/African American	6.5% (8)	Black/African American	0% (0)
Asian	23.4% (29)	Asian	17.2% (5)
Multiracial/Other	6.5% (8)	Multiracial	3.4% (1)
Current Gender Identity		Current Gender Identity	
Woman	93.0% (107)	Woman	90.6% (48)
Another gender identity (Man, Non-binary, other)	7.0% (8)	Another gender identity (Man, Non-binary, other)	9.4% (5)
Sexual orientation		Sexual orientation	
LGBQ+	24.3% (28)	LGBQ+	27.6% (8)
Heterosexual/Straight	75.7% (87)	Heterosexual/Straight	60.1% (18)
Unknown	0% (0)	Unknown	3.4% (1)
Classification		Age	
Freshman	9.0% (10)	18–20	20.7% (6)
Sophomore	21.6% (24)	21–22	41.3% (12)
Junior	27.0% (30)	23–25	17.3% (5)
Senior	23.4% (26)	26+	20.7% (6)
Graduate Student/Other	18.9% (21)		

### Data Analysis

Quantitative analysis of initial and follow-up surveys involved descriptive and bivariate analysis, including measures of central tendency, frequencies, paired *t*-test, and regression analyses. Longitudinal data were linked between baseline and 6-month follow-up assessment, with only complete cases analyzed in paired analyses. Data on individuals were independent, within pair differences were asymptotically normally distributed, and there were no outlying values; therefore, paired *t*-tests were utilized to test for within person over-time change for each of the measures. All percentages reported reflect the proportion of the sample responding to the specific item or items under analyses. Regression models were used to estimate the relative associations of safety indicators on violence and academic impacts. Linear models were used for continuous outcomes and ordered Logistic regression models were used for the categorical outcome GPA. In all cases, models were tested for and met distributional assumptions. The Wald test, measured as the estimated regression beta/SE, was utilized to test for statistical significance of association.

Qualitative data were analyzed using approaches associated with thematic analysis (Braun & Clarke, 2020, 2021) as a strong approach for understanding experience, process, and generating understanding for applied research. In this approach, the first and last author first *familiarized* themselves with the data through reading transcripts, reviewing memos, and listening to audio files. From that review, initial inductive information about campus advocacy was developed from participants’ own perspective and the previous research findings from earlier project stages were expanded on for analysis. An initial codebook related to campus advocacy experience was developed from this process. Then the authors embarked on *coding* with a flexible, iterative codebook of 24 codes from 12 categories was developed related to CBA program impacts. Data were coded by the first, third, and last authors. *Initial themes* were generation from coding through analytic and reflexive memos, analysis meeting, and secondary relational coding related to the experience and outcome of campus advocacy. After initial theme development, a second phase of coding was employed across the full dataset to further *develop themes* with a focus on CBA programs address academic and safety concerns. Quality criteria include reflexive memoing (Braun & Clarke, 2021) and a credible, resonance, ethical approach (Tracy et al., 2010). The refined themes were named and integrated with quantitative data for the analysis below (Creswell & Plano-Clark, 2017).

## Results

This study seeks to understand the steps CBA programs use to mitigate safety and academic challenges faced by students, as well as begin to build knowledge related to their success in addressing these dual impacts of violence. Using a mixed-methods analysis of longitudinal surveys and qualitative interviews focused on data related to safety and academics, it uncovered several findings of note in response to both research questions. Primarily qualitative data help to explicate the ways that CBA programs work to address safety and academic concerns, whereas qualitative and quantitative data come together to highlight student outcomes. Substantial safety and academic-related changes were observed, and analysis of qualitative interviews illuminated the mechanisms through which CBA programs may positively influence student safety and academic outcomes.

### How Do Campus Advocacy Programs Address Survivor Safety and Academic Concerns?

**Figure 1.**
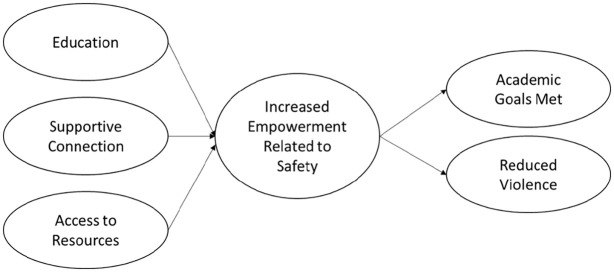
The process of campus-based advocacy.

Qualitative analysis of 29 semi-structured interviews exposed mechanisms by which advocacy programs address safety and academic needs. A primary focus of CBA is improving survivor safety through education, resource provision, and supportive connections. In summary, student-survivors shared that CBA provide a holistic approach to survivor well-being that has cascading safety and academic impacts. CBA services focus on multidimensional aspects of safety, including physical (e.g., violence), emotional (e.g., mental health), structural (e.g., built environment), and social (e.g., peer network) aspects that are of concern after violence and harm. Participants indicated that engagement with CBA programing focuses on education, supportive connection, and access to resources and leads to increased empowerment related to safety, which in turn positively impacted their academic outcomes, and increased safety. This process is illustrated in Figure 1. Below, these factors are exposed with participant description.

### Education

Education about violence and trauma provided within the context of CBA services helped students make meaning of their situation. Education included a focus on health impacts, information about consent, and uncovering patterns of power and coercive control in relationships. One survivor shared how this helped them process and identify the violence they were experiencing:

The insight and being able to talk to <advocate> and tell what my ex is doing and everything that was going on, and <advocate> being like, “this is abusive behavior. This is what this is.” This is what we know and doing it from that space was helpful for me. It felt validating and it felt- I felt like I knew better what was going on and it also gave me the opportunity to admit to myself that I was going through that. P15

This process of making sense of experiences also helped survivors reduce self-blame, facilitating communication about their experiences and needs with less attached shame. This led to meeting immediate needs as well as longer-term stability.

One thing that me and my advocate had talked about that made me think about my experience really differently was that we talked about how I felt really shameful about the experience, but then we kind of worked through shifting the shame and taking that guilt off of me. That made me feel like I wasn’t the one in the wrong, so that made me—I think that was one of the reasons that made me—that made it really hard for me and affected my mental health the most, so working through that had helped a lot, and it impacted everything else. P19

CBA services also supported survivor education around managing violence and trauma impacts and strategies to help identify and address potentially harmful situations and manage threats to safety. Participants identified skills related to increased education included setting boundaries, improving support networks, sharing emotions, and accepting needed resources. One participant linked this increased education about safety risks to empowerment to their future:

I think whenever I come across myself feeling like I’m getting into a serious situation, I feel like I have the steps now to just get out of it before it gets bad. I feel like that definitely made a difference because I’ve experienced feeling helpless before, and now whenever I’m in a situation that felt similar to what I’ve gone through, I feel like I’m in power, and I can protect myself . . . I think my main goal was to get powerful or feel more powerful, and I think that was definitely something I achieved just throughout the services and then my experience at <university> after that. P21

Learning and setting boundaries was especially significant for many CBA service users, given both their developmental stage as young adults and the loss of power and control that typically accompanies experiencing interpersonal violence. Many participants linked education about violence and safety and ability to set boundaries related to relationships.

I do think especially being involved and be working in <program> really helped me understand more about myself and my boundaries, and it’s okay to put boundaries. People have to respect them, right, and to understand more about consent, and what is consent, and what isn’t consent ’cause I think before that, I was—I would make a lot of excuses for people, right. Now, I know that like, “Oh, well, you were the one that didn’t respect me, not the other way around.” P29

For a few participants, education on safety planning skills like identifying supports and self-defense mechanisms were not perceived as being helpful. For example, one survivor shared

They basically just told me, like I said, the different numbers I can call, and if I do become certain that someone is stalking me, whatever, I would just make sure I was with somebody or carry pepper spray on me and just constantly I’d be looking at my surroundings. P4

### Supportive Connections

Along with learning information and skills, the impact of CBA services for many study participants was a feeling of safety facilitated by their supportive connection with the advocate. One participant talked about the way that support from their advocate was helpful both when they were managing their situation on their own, and as they were making choices about institutional processes. They shared “*I did feel very supported, and when I would ignore it, or report it, or whatever I chose to do, I felt like I had someone on my side that was rooting for me.”* P15. Another participant reflected on how advocate outreach was central to their experience, sharing:

I definitely was grateful that there was something <advocacy> in place for me and especially the fact that they reached out to me. That helped me a lot, and I was super appreciative of that ’cause it’s not like a situation happened and then I had to forget about it. It was actually like I got to be able to talk through it and feel okay after it. P21

Along with serving as a source of connection and support, CBA services helped students build, rebuild, and expand their own support networks, which students expressed improved their emotional and physical safety. One participant shared how working with the program helped them connect with others around them, stating:

I was at a loss for words, and I was telling my close friends about it. After receiving those services, I felt a little bit better or I guess more assured in how I was explaining this to someone else because sometimes they might not understand that. Definitely seeing a professional really helped me communicate that to someone else more effectively. P11

The support provided by advocacy programs facilitated a greater sense of safety on campus at large by providing micro-level supports for survivors, but also a macro-level signal that the campus takes violence seriously enough to have dedicated services to address the impacts of violence. As one participant shared about their advocacy experience, “*My freshman year going to <university> thinking that it was just gonna be such a big school, and I was gonna feel kind of alone, that was not the issue at all. I felt so welcomed and so understood by <*advocacy program*>.”* P5. One survivor highlighted the link between the micro support of advocates and the sense of the institution supporting them:

I think I was definitely on the track to having my whole college experience be very negative. If it wasn’t for advocacy services, I think I probably would have hated my entire college experience because it would have just felt like full failure and loss and more out of my control than what would feel manageable. And so because of that, it just makes me feel less angry at <university> as a whole. P16

CBA support made survivors feel their university takes their experiences seriously, and that in turn made them feel like they and their peers were more worthy of support. This propelled students to share resources with others, further strengthening the campus environment:

I think now the university takes its students seriously and especially in terms of harm, in terms of whether it’s physical or mental. I think that’s good because it makes me feel a bit more safe on campus because especially as a woman, you get afraid of what’s can happen. Now that I'm aware of all those things I'm more comfortable, and I can also acknowledge or tell other people about those services too are necessary. P6

However, a few survivors say CBA programs as a part of a larger strategy of avoidance, rather than as a sign of support from the university:

It just made me realize that the university doesn’t really care about the students. They just wanna keep them around for another tuition that they can charge the students. You know what I mean? Regardless of what kinda violence acts they do upon other students. Like I said, in a way, I felt <advocacy> is just there to hide that so that, if a student keeps feeling bad, depressed like I did, crying during class like I did because they wouldn’t remove the student from campus, they can just put a band-aid over it with <advocacy program>. P3

### Access to Resources

A third factor discussed by student-survivors as impactful centered on resource access. CBAs assisted service users in identifying their civil and legal rights and connected them to campus and community-based resources that address immediate and longer-term needs after experiences of violence. One survivor summed up the wide range of resources thusly:

<Advocate> pointed me to a free legal aid service in case I was to need legal support, I think. . .She also provided guidance for the crime’s victims also for the family violence center. I think the center for—something about family and sexual violence, I believe. She also helped me with my grades to see if I needed to go with the accommodation center at the university. I think she gave me the link to immigration advising because I’m [an] international student. P27

Advocates offered law enforcement services as a safety resource for survivors to consider. For some survivors, use of law enforcement and other criminal justice supports improved safety. One survivor described the decision to engage with law enforcement.

And because I was feeling emotionally a little bit more supported, I was willing to do the research and see if any laws were violated and if so, is it worth pursuing? And there was a clear violation of the law. And so I was like, you know what, I should just … I felt motivated to prevent this from happening to someone else. And so I went through with it at [the police department]. P16

One of the most critical sets of resources provided by CBAs center on supporting academic goals and assisting with accommodations. A survivor noted the wide range of academic resources available through CBAs, and how they were able to use those supports to enhance their wellbeing, further enhancing their safety and academic outcomes:

So the first time when they reached out to me, I didn’t even realize what they could do for my benefit. And I was told that they could help me communicate with my professors about lightening a workload or making sure that I was in touch with<service>and things like that. And I was really, really grateful because I didn’t even know that those options were available to me. And I was preparing myself to fail a semester or just skate through it. And most of my professors were very kind and accommodating . . . And so it became more collaborative and that way it was really, really wonderful. P16

Advocates helped assist service users to obtain academic accommodations and, in some cases, disability-related accommodations related to trauma-impacts. Nearly all interviewed participants were offered help with accommodations. For most participants, accommodations such as extensions, class withdrawals, alternative attendance arrangements, and extra time on assignments helped them to meet their academic goals. One survivor noted the importance of getting help with “*My class registration, definitely. Getting me extensions and accommodations from my professors. Also, I ended up withdrawing for a little bit from school, and she was really helpful in even suggesting that as an option to me.”* P1. Along with addressing academic needs, academic accommodations were identified as a resource for addressing safety needs, especially in cases of dating violence and stalking.

Every semester, when I registered, <advocate> would compare my schedule to the schedule of the person that harmed me because he was in the same major as me. Obviously, she wouldn’t tell me what classes he was in and stuff, but she would say, “Oh, I’m looking at your schedule right now. You might want to think about changing your philosophy discussion section” or something like that. That was really helpful to me because it was like a very small major that we were in. P1

Notably, for some participants academic accommodations were not utilized or deemed unnecessary. For example, one survivor shared that “*I think I was okay academic wise. I’m the kind of person that compartmentalizes, so that wasn’t the big issue for me. I think the biggest issue was just fighting my inner demons and thoughts.”* P21.

Through education, supportive connections and resources, student service-users reported feeling more empowered and in control, which lessened their safety concerns. They also reported feeling more comfortable and confident in identification of potential harms, in using strategies to improve physical and emotional safety threats, and in building a supportive web of informal and formal connections. One survivor shared how this impacted them, stating, “*I feel like I finally got my life under control.*” P5 and another shared “*Just like having that support and people who honestly believed me, it was really helpful. It made me feel like I could regain some control.”* P16. This is also reflected in longitudinal survey data, as participants were asked about their sense of safety related empowerment, and their sense that their community could positively contribute to their safety. Participants generally reported confidence in their internal tools for safety and reported confidence in their community’s ability to support them. Across time points, participants had broadly positive views of their internal tools for addressing threats of violence and their expectations of community support, with mean scores at 3 (out of 4), representing a sense that the statements are ‘somewhat true’ for them. These feelings were stable between initial assessment and 6-month follow-up.

### What changes are observed in safety and academics for student-service users over 6 months?

#### Safety

Participants endorsed a high level of violence exposure since enrollment at their current university, with 59.5% reporting sexual violence victimization, 81.5% reporting stalking victimization, and 55.4% reporting IPV victimization since enrollment (see Table 2). Across forms of violence, statistically significant reductions were observed in frequency and severity of sexual, stalking, and IPV from baseline to follow-up, with only 5.5% of participants reporting any sexual violence experiences, 50% reporting exposure to stalking victimization, and 15.1% reporting exposure to IPV 6 months after the initial impact survey. Among participants in the matched sample, 56.8% reported experiencing at least one incidence of school sabotage between enrollment and initial impact survey, whereas only 17.5% reported experiencing school sabotage in the 6 months between the first survey and follow-up.

**Table 2.** Safety From Interpersonal Violence Since Enrollment and Since Initial Impact Survey.^a^

*n* = 115 Endorsement in Sample	Since Enrollment (Initial Survey)	6-Month Follow-Up	
Sexual violence	59.5%	5.5%	
Endorsed at least one item			
Stalking	81.5%	50.0%	
Endorsed at least two items			
Intimate partner violence	55.4%	15.1%	
Endorsed at least one item			
School sabotage	56.8%	17.5%	
Endorsed at least one item			
Assessment of Scale changes	Mean (*SD*)	Mean (*SD*)	*p*-Value^b^
Sexual Violence Scale	4.03 (1.79)	3.12 (0.65)	<.001
Stalking Scale	13.63 (6.44)	9.65 (4.96)	<.0001
Intimate Partner Violence Scale	9.21 (4.60)	6.51 (2.02)	<.001
School Sabotage Scale	11.66 (7.06)	8.82 (2.22)	<.001

a Matched sample between baseline and follow-up 2, *n* = 115.

b Two sided paired *t*-test.

Potential differences in violence reduction were explored by survivor racial identity (White/not White) and sexual orientation (lesbian/gay/bisexual/queer identified/straight/heterosexual). No statistical differences were observed in the extent of change in stalking, sexual, or IPV or school sabotage from initial survey to follow-up by participant race or sexual orientation. Based on the qualitative findings, a series of regression models were run to understand the association between stalking, IPV, and school sabotage at follow-up and safety and empowerment related to safety at baseline (see Table 3). Across all three outcomes, the level of violence reported at initial assessment is significantly associated with the level reported at follow-up. Those experiencing more violence at initial assessment were still experiencing higher levels at follow-up. For models assessing the extent of IPV and school sabotage, higher self-reported internal tools for addressing violence at baseline were associated with reductions in violence at 6-month follow-up.

**Table 3.** Regression Models for the impact of Internal Tools, Expectations of Support, and Extent of Violence at Baseline on Extent of Three Forms of Violence Reported at Follow-Up.^a^

Measure	Stalking		IPV		School Sabotage	
	Beta (*SE*)	*p*-Value	Beta (*SE*)	*p*-Value	Beta (*SE*)	*p*-Value
Internal tools	−.17 (0.32)	.60	−.32 (0.13)	**.02**	−.43 (.16)	**.01**
Expectations of support	−.19 (0.40)	.63	.23 (0.16)	.15	.31 (0.20)	.12
Initial assessment of outcome	.34 (0.11)	**<.00**	.21 (0.05)	**<.00**	.19 (0.05)	**<.00**

*Note*. IPV = Intimate partner violence.

a Sexual violence was not included due to lack of variation at follow-up.

Bold values are statistically significant.

#### Academic Experiences

Participants reported reductions in academic disengagement behaviors from initial survey to follow-up, with statistically significant reductions in missing class due to abuse, being unable to attend class due to safety concerns, being unable to attend class due to mental health concerns, missing exams or quizzes, and sleeping in class (see Table 4). Participants also reported a statistically significant overall reduction in academic disengagement (*t*[84] = −3.68, *p* = .001).

**Table 4.** Academic Disengagement.

(Matched Sample *n* = 115)			
	Initial Survey Mean (SD)	Follow-Up Mean (SD)	*p*-Value^a^
Missed class (because of abuse, violence, or harassment experiences)	2.14 (1.16)	1.53 (.85)	**<.00**
Unable to attend class for safety concerns	1.64 (1.01)	1.30 (.74)	**.01**
Unable to attend class for MH Issues	2.60 (1.17)	2.14 (1.20)	**.01**
Missed an exam, quiz, or other graded assignment	2.32 (1.12)	1.77 (1.00)	**<.00**
Attended class intoxicated or “high”	1.22 (.67)	1.06 (.28)	.09
Slept in class	1.94 (1.03)	1.56 (.90)	**.00**
Failed in class	1.62 (.88)	1.38 (.86)	.15
Dropped a class	1.73 (1.09)	1.44 (.80)	.06
Thought about quitting school	2.42 (1.34)	2.30 (1.26)	.46
Turned in homework/assignment late or not at all	2.36 (1.08)	2.10 (1.16)	.16
Withdrew for a semester	1.19 (.61)	1.08 (.51)	.07
Full scale	21.18 (7.27)	17.71 (6.63)	**<.001**

a *p*-Value from paired *t*-test of change from initial assessment to follow-up assessment (scale range 11–44).

MH = Mental Health.

Bold values are statistically significant.

In terms of self-reported academic outcomes, a greater percentage of participants reported having an “A” GPA at follow-up (64.2%) compared to the initial survey (56.5%), whereas a lower percentage reported “B” and “C” GPAs at follow-up than at the initial survey. When asked if their GPA was higher, lower, or about the same as last semester, 28% of participants said it was higher at the initial impact survey, whereas 36% said it was higher at follow-up. Comparatively, 26% of participants said they had a lower GPA than last semester at the initial survey, whereas only 18% said they had a lower GPA than last semester at follow-up.

To assess the role of academic impacts of violence and empowerment related to safety on student self-reported GPA, an ordered logistic regression was run. Explanatory variables were taken from initial impact data, and included academic disengagement of violence scale mean, internal tools for addressing violence scale mean, expectations for community support addressing violence scale mean, and GPA (A, B, C, D, & F GPA) at initial survey. The dependent variable was self-reported GPA at follow-up (see Table 5).

**Table 5.** Impact of Initial Empowerment Related to Safety, Academic Disengagement, and GPA on Follow-up GPA for Campus-Based Advocacy Service Users.

Student GPA (follow-up)			
	beta (*SE*)	95% CI	*p*-Value
Internal tools	.10 (0.12)	[−0.13, 0.34]	.40
Expectations of support	−.46 (0.18)	[−0.80, −0.12]	**.009**
Academic disengagement behaviors at baseline	.14 (0.05)	[0.05, 0.23]	**.003**
GPA at baseline	2.26 (0.51)	[1.26, 3.25]	**<.001**

*Note*. GPA = grade point average.

Bold values are statistically significant.

Student expectations of community support, extent of academic disengagement, and GPA at baseline were all associated with GPA at follow-up in this model. Students with higher expectations of community support at the initial survey had higher GPAs at follow-up, whereas greater extent of academic disengagement at initial survey was associated with lower GPA at follow-up. Participants shared how this stability and healing led to a cascade of other positive outcomes, including increased safety, and improved academic outcomes. As one participant shared “*I thought that because of personal trauma, I would have to experience loss and in all other aspects of my life. And I think through advocacy services, that loss was greatly reduced. I didn’t lose my academic standing*.” P16 Further, for nearly all participants interviewed, CBA services contributed, at least in part, to greater stability and healing after violence. As one survivor put it:

I would say that <university> helped me a lot to become stronger mentally and acknowledge that that was abuse. I would say I’m not completely healed from that, but I am definitely so, so much stronger, and so much better off now. P5.

## Discussion

High rates of IPV, school sabotage, stalking, and sexual assault in college student populations necessitate a robust prevention response to eliminate violence as well as a powerful intervention response to mitigate impacts on students. Survivors of violence on college campuses are particularly vulnerable to revictimization and negative academic outcomes. The results of this mixed-methods study of CBA services on the safety and academic needs of student survivors demonstrates the potential for these services to make positive impacts on college campuses. Data from qualitative interviews helped to identify the potential pathways through which CBA programs support survivors, address needs, and reduce experiences of future violence and academic disengagement. Student survivor experiences demonstrate that CBA services improve survivor safety through (a) education; (b) supportive connection, and (c) access to resources. These three pillars of CBA support survivors in enhancing their tools for addressing violence and in facilitating a sense of empowerment to address concerns and needs. Increased empowerment related safety leads to reduced exposure to violence and reduced risks for violence, as well as re-engagement in academic goals and improvement in outcomes. Longitudinal quantitative analysis of survivor data highlighted that service recipients experienced reductions in violence exposure across forms of violence over time, with only 5.5% of survivors reporting experiences of sexual violence at 6-month follow-up, compared to 59.6% in the initial survey, along with improvements in academic disengagement for student service users at 6-month follow-up as compared to the initial survey. However, without a true baseline or comparison group, it should be noted that these changes may reflect normal changes in violence exposure over time, and that causation cannot be established.

Previous research has demonstrated that community-based advocacy programs enhance survivor empowerment, a critical component of longer-term safety and well-being (Goodman et al., 2015). The work of Goodman et al. (2015) highlights domains of survivor empowerment that include internal tools (e.g., self-confidence related to ones’ own ability to manage one’s safety situations) and expectations of support (e.g., the extent to which you feel your community can and will provide the support you need for safety). Longitudinal data reflect that both student-survivors’ internal tools and expectations of support play crucial roles in outcomes after experiences of violence. Improvements in safety from violence were linked with survivors’ sense of their internal tools for responding to safety threats, whereas students’ expectations of support on campus were linked with improved academic outcomes at 6-month follow-up.

These findings indicate that advocates who work with survivors to build skills in safety planning, practice safety skills and coping strategies, and work with survivors to build up their own internal tools to support safety may be positively impacting safety from violence. Thus, CBA could be considered both an intervention, a restorative act, and a component of a prevention effort to curtail future violence for the student and campus. Importantly, stalking rates remained higher than other forms, and was experienced at follow-up both by survivors who sought CBA services for stalking initially, and by survivors seeking services for other forms of victimization. As noted elsewhere (Logan & Walker, 2017), stalking is a form of violence that is often difficult to address, especially when it involves technology and third parties, and thus merits further consideration.

CBA services aim to build student survivors’ expectations that the advocacy program is there for support, and advocates’ effective navigation of campus systems enhances survivors’ campus and community engagement, which was directly linked to improved academic outcomes. This increased trust in and engagement with the community is associated with increased survivor GPA at follow-up compared to initial assessment. For students in higher education, addressing and mitigating academic impacts of violence takes on a crucial role in effective CBA experiences, as long-term economic and developmental outcomes are linked to sustaining in and completing degree programs or training courses. More work is needed to understand the full impact of experiencing interpersonal violence during higher education on academic and ultimately career and economic outcomes. However, this study sheds important light on the extent of school sabotage experienced by students and the role of CBA in providing effective academic safety planning, reducing academic impacts and improving survivor outcomes.

Study findings mirror results in community-based advocacy research, supporting the use of a trauma informed, (student) survivor-centered, voluntary, and culturally adaptive model in reducing revictimization risks and increasing economic and educational outcomes after violence (Wood et al., 2021; Rivas et al., 2015). While this sample and previous research in this university system both highlight that survivors of color and survivors who identify as part of the LGBTQ+ community face higher rates of violence victimization, no differences were observed between students who identified as lesbian/gay/bisexual/queer and students who identified as straight/heterosexual, nor between students of color and students who identified as white in terms of change in safety outcomes from initial survey to follow-up. This should be considered in the specific context of our research. Survivors in our study chose to engage with CBA services at least once and chose to participate in evaluation research on the program. Additionally, the programs studied all put central focus on the needs of students with diverse identities, including prioritizing a diverse staff that reflects the student body of each institution, and partnering or co-locating with programs that support students of color and the LGBTQIA+ communities on campus.

## Limitations

Several limitations deserve note in light of study findings. First, while the study used longitudinal methods, it is important to highlight the lack of randomization or a true baseline design. Because participants self-select into advocacy services (which is crucial to a voluntary service model), and because they were contacted to enroll in the study after they began receiving CBA services, the study cannot capture the full cause–effect relationship between services and survivor outcomes. Estimates of program impact are also less precise because of the choice to enroll participants after initial service engagement. There were some participants in the longitudinal survey who were lost to follow-up and not included in these matched analyses, which could impact the overall study findings. However, a retention rate slightly over 80% is considered acceptable given the population and challenge of retaining survivors in longitudinal work (Hanna et al., 2015). All programs involved in the study are located within a single university system in the United States, strengthening the evidence for effectiveness in this context, but threatening generalizability to other university systems or state that have differing student contexts, needs, and policies. Data were also entirely self-reported, including such information as GPA and course engagement, which could be captured in multiple ways. While this supports the central role of the student-survivor’s perception in the work of CBA services, future work could be strengthened by gathering multiple strands of outcome data on issues such as GPA, retention, or course load from university sources where it is available.

## Implications

CBA programs positively impact student-survivors safety and academic outcomes, and thus the overall climate and efficacy of their hosting institutions. While the prevalence of such programs on college campuses in the United States in unclear, it has been more clearly established that they are often understaffed where they do exist (Klein et al., 2023). Without proper staffing, programs will be less able to engage in the time-consuming process of individual survivor advocacy that has been shown to lead to positive safety and academic outcomes. As such, program and campuses should emphasize institutional, financial, material, and technical support to provide programs with robust resources to fund staff as well as provide needed materials and service for survivors. Depending on the context and needs of an individual campus, this may include access to campus-based housing, food, cash, and financial assistance, mental health professionals internal to the CBA program or closely aligned in other campus programs, as well as competitive pay and benefits for program staff. Given the role of CBA programs in retention and safety of students, campuses should view these investments as high priority and low risk (Klein et al., 2016).

Previous work has highlighted the central role of confidentiality in effective survivor services, and the challenges that university, state, and federal policy can pose in providing truly confidential support to survivors on campus (Javorka & Campbell, 2019). This study highlights the potential for impactful services in a campus context. It is important to note that each of the studied programs aims to provide confidential services, includes frequent discussions of the limits of confidentiality and the role of policy as part of their CBA services, and collaborate with community based IPV and SV services providers. Such collaborative relationships can be crucial for ensuring effective CBA services (Javorka & Campbell, 2019).

Several areas for future research are underscored by these findings. Work should expand in scope and rigor, with research in programs across a range of university settings, geographic contexts, and student populations that incorporate a full baseline and other rigorous approaches. Diverse settings are needed to better understand the role and impact of cultural and contextual adaptations in services for survivors, particularly survivors of color and members of the LGBTQIA+ community who face increased risks of violence due to discrimination, barriers to service access, and other systemic failings. Investigations of the impact of CBA programs on outcomes including well-being and social connectedness on campus is warranted, given the potential for this service to be a marque approach for supporting student survivors.
